# Molecular Cloning, Sequence Characterization and Expression Analysis of a CD63 Homologue from the Coleopteran Beetle, *Tenebrio molitor*

**DOI:** 10.3390/ijms141020744

**Published:** 2013-10-15

**Authors:** Bharat Bhusan Patnaik, Seong Min Kang, Gi Won Seo, Hyo Jeong Lee, Hongray Howrelia Patnaik, Yong Hun Jo, Hamisi Tindwa, Yong Seok Lee, Bok Luel Lee, Nam Jung Kim, In Seok Bang, Yeon Soo Han

**Affiliations:** 1 Division of Plant Biotechnology, Institute of Environmentally-Friendly Agriculture (IEFA), College of Agriculture and Life Sciences, Chonnam National University, Gwangju 500-757, Korea; E-Mails: drbharatbhusan4@gmail.com (B.B.P.); ndnd2@nate.com (G.W.S.); lhjzzdd@hanmail.net (H.J.L.); howrelia@yahoo.com (H.H.P.); yhun1228@chonnam.ac.kr (Y.H.J.); tindwa@yahoo.com (H.T.); 2 National Research Laboratory of Defense Proteins, College of Pharmacy, Pusan National University, Jangjeon Dong, Kumjeong Ku, Busan 609-735, Korea; E-Mails: kangseongmin85@gmail.com (S.M.K.); brlee@pusan.ac.kr (B.L.L.); 3 Department of Life Science and Biotechnology, College of Natural Sciences, Soonchunhyang University, Asan city 336-745, Korea; E-Mail: yslee@sch.ac.kr; 4 Division of Applied Entomology, National Academy of Agricultural Science, Rural Development, 61th, Seodun-dong, Gwonseon-gu, Suwon, Gyeonggi-do 441-853, Korea; E-Mail: vastnj@korea.kr; 5 Department of Biological Science and the Research Institute for Basic Sciences, Hoseo University, Asan 336-795, Korea; E-Mail: isbang@hoseo.edu

**Keywords:** molecular cloning, CD63, TM_4_SF, expression analysis, *Tenebrio molitor*, immune elicitors

## Abstract

CD63, a member of the tetraspanin membrane protein family, plays a pivotal role in cell growth, motility, signal transduction, host-pathogen interactions and cancer. In this work, the cDNA encoding CD63 homologue (TmCD63) was cloned from larvae of a coleopteran beetle, *Tenebrio molitor.* The cDNA is comprised of an open reading frame of 705 bp, encoding putative protein of 235 amino acid residues. *In silico* analysis shows that the protein has four putative transmembrane domains and one large extracellular loop. The characteristic “Cys-Cys-Gly” motif and “Cys188” residues are highly conserved in the large extracellular loop. Phylogenetic analysis of TmCD63 revealed that they belong to the insect cluster with 50%–56% identity. Analysis of spatial expression patterns demonstrated that TmCD63 mRNA is mainly expressed in gut and Malphigian tubules of larvae and the testis of the adult. Developmental expression patterns of CD63 mRNA showed that TmCD63 transcripts are detected in late larval, pupal and adult stages. Interestingly, TmCD63 transcripts are upregulated to the maximum level of 4.5 fold, in response to DAP-type peptidoglycan during the first 6 h, although other immune elicitors also caused significant increase to the transcript level at later time-points. These results suggest that CD63 might contribute to *T. molitor* immune response against various microbial pathogens.

## 1. Introduction

The tetraspanin superfamily of proteins, first recognized in 1990, has emerged as the organizer of functionaries of cell-surface proteins because of their ability to cross-talk with certain other signaling and adhesion molecules involved in cell differentiation. They function in the critical role of “molecular facilitators”, assembling larger molecular complexes and providing them with stability assisting in working in a more orderly manner and efficiently. The first protein belonging to the family, ME491/CD63, was characterized in 1988, and the hallmark protein sequence motifs were reported in 1990 [1,2]. Tetraspanins were originally identified as tumor antigens and are broadly expressed as integral membrane proteins with as many as 33 members in humans, 37 in insects (*Drosophila*), 23 in sea anemones (*Nematostella*) and 17 in plants (*Arabidopsis*) [3–6].

The conserved predicted structure spans four hydrophobic, putative transmembrane domains (TM1–TM4), forming a small and a large extracellular loop (EC1 and EC2) with short intracellular amino and carboxyl tails, and therefore, called TM_4_SF proteins. In addition, 4–8 highly conserved extracellular cysteines have been known, of which two are present in a CCG motif located 28–47 residues after third transmembrane domain [7]. The four cysteine residues generate a defining mushroom like structural signature for tetraspanins large extracellular loop, mediating specific protein–protein interactions in the tetraspanin web [8,9]. The tetraspanin superfamily comprises four subfamilies: the CD-non63, CD63, uroplakin and RDS, of which CD63 has the most ancient origin, mostly exerting their function through interaction with integrins at “tetraspanin-enriched micro domains” (TEMs) or “tetraspanin web” at the cell surface [9–12]. Tetraspanin proteins have been known to be synthesized in the endoplasmic reticulum (ER) and after palmitoylation are subsequently transported to the cell surface as building blocks of TEMs [13]. They also have promiscuous presence in liposome-related organelles or secretory lysosomes, fusing with the cell surface, releasing their content into the extracellular environment [11,14].

CD63 encoded protein is a cell surface glycoprotein that is known to complex with integrins such as β1 integrins [15], other tetraspanins e.g., CD81, CD82, CD9, CD151, and CXCR4 [16], kinases [15], adaptor proteins [17] and other proteins including L6 antigen [18], syntenin-1 [19], TIMP-1 [20], HK-ATPase [21] and MT-1-MMP [22]. These myriad functions have led to the suggestion that CD63 may function as “adaptor proteins” [23], which organize the relative position of other cell-surface molecules and modulate their function. CD63 was identified as a platelet-activating antigen, originally known as platelet glycoprotein 40 or melanoma antigen 491 [24].

CD63 has been found in many types of blood cells and endo/epithelial cells such as dense granules in platelets, α-granules in megakaryocytes, cytotoxic T-cell granules in T-cells, azurophic granules in neutrophils, Weibel-palade bodies in vascular endothelial cells and eosinophilic granules in B cells, dendritic and epithelial cells [25–28]. Upon cell activation, CD63 is mobilized to the cell surface via the exocytic pathway or to endosomes via intracellular pathway and gets involved in various immunophysiological processes [12,29]. On the other hand, it activates protein-tyrosine kinase (PTK) and enhances the PTK-induced inhibition of ROMK channels [30]. CD63 also may represent an important new therapeutic target for the development of anti-retroviral drugs as it has been shown that down-regulation of the gene leads to reduced production of HIV protein Tat and also inhibits the production of late protein p24 [31]. It has also been reported that CD63 is a critical mediator of viral oncogene, latent membrane protein 1 (LMP1), which functions inside and outside infected (tumor) cells by limiting constitutive activation of NF-κB through promotional trafficking in the endosomal-exosomal pathway [32]. Reduction in CD63 expression, a marker identified in the malignant progression of human melanoma contributes to invasive and metastatic ability of human melanoma cells [33,34].

CD63 cDNA has been identified, characterized and studied for its expression in the channel catfish*, Ictalurus punctatus* and has shown 52%–55% identity among fish counterparts and only 43%–46% identity among mammalian counterparts with higher expression in intestine and anterior kidney [35]. Moreover, Liu *et al.* [36] reported the cDNA encoding CD63 from gut cDNA library of amphioxus, *Branchiostoma belcheri tsinglauense* and found it to be extremely close to vertebrate CD63 with the transcript found abundantly in muscle, ovary and foregut. CD63 was cloned from the Chinese shrimp, *Fenneropenaeus chinensis* and the transcript was found to be expressed in nerves, epidermis and heart with no expression in intestine, muscle and lymphoid organ. The same was also found to be upregulated when challenged by live white spot syndrome virus (WSSV) and heat-inactivated WSSV [37].

The biological role of tetraspanins in cellular dynamics has been well established, but there are no reports about their functional role in beetle immunity against microbial elicitors. *Tenebrio molitor* is a species of darkling beetle and is a resourceful model for studies in biochemistry, immunology and physiology [38–40]. It is an efficient laboratory insect because of its larger size, ease of handling and culture throughout the year. In order to gain insight into the innate immune mechanism of insects, we screened immune-related genes in *T. molitor* by an expressed sequence tag (EST) study [41]. We found clones that correspond to the partial sequence of CD63 in *T. molitor*. In a larger perspective, we wanted to study the specific interactions of *T. molitor* CD63 with microbes and their cell wall components. For the same reason, we made an initial attempt to clone the full-length cDNA (TmCD63), and characterize the sequence in detail using bioinformatics and experimental approaches. In addition, we examined the developmental and tissue-specific expression profiles of the gene at the basal level. We also report the expression profile of the gene after stimulation of *T. molitor* larvae with PGN and β-1,3 glucan as well as *Acholeplasma* and *Listeria monocytogenes* infection.

## 2. Results

### 2.1. Bioinformatics Analysis of TmCD63 and Structure of Deduced Protein

A full-length cDNA sequence encoding the first CD63 from *T. molitor* larvae was cloned and subsequently found to show high similarity with vertebrate CD63, and therefore nominated as a putative homologue in the model organism. Initially, the partial sequence of the putative *CD63* gene was identified by conducting a comprehensive expressed sequence tag (EST) analysis of *T. molitor* larvae towards identification of immune-related genes [41].

**Figure 1:**
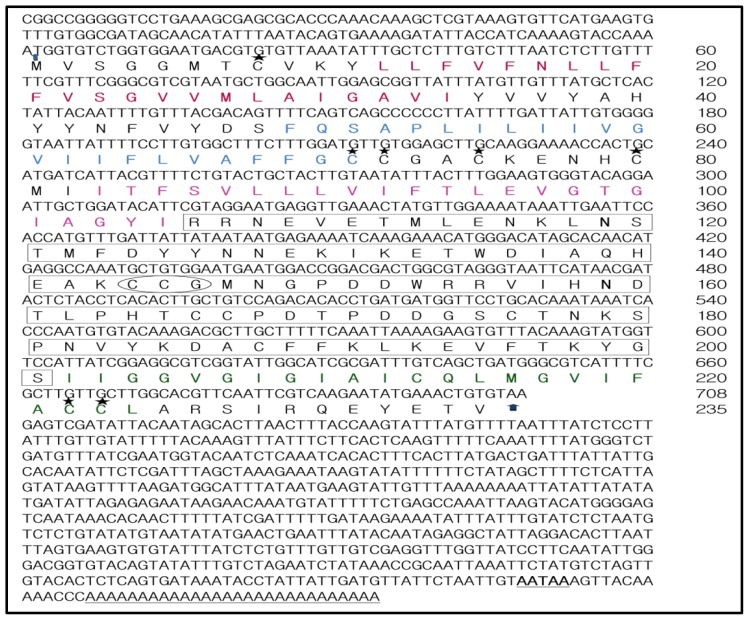
Nucleotide and deduced amino acid sequence of cDNA encoding TmCD63. Deduced amino acids are denoted as a one-letter code, arrows indicate initiation codon and the translation stop codon. The poly-A signal in 3’-UTR is bold and underlined, whereas the poly-A tail is underlined. The conserved TM regions 1–4 are represented in red, blue, pink and green respectively. The large extracellular loop (LEL) is boxed and contains the conserved “Cys-Cys-Gly” motif (encircled). The predicted palmitoylation cysteines are indicated in five-point star format and predicted *N*-glycosylation arginine residues are indicated in bold.

The full-length cDNA of TmCD63 comprises 1547 nucleotides with an open reading frame (ORF) of 705 bp, beginning with the initiation sequence “ATG” and ending with “GTG”, before the “TAA” stop codon. The cDNA contains a 5′ untranslated region (UTR) comprising of 120 nucleotides and a long 3′-UTR containing 719 nucleotides (Figure 1). A putative polyadenylation consensus signal (AATAA) was seen 14 nucleotides upstream of the 28 bp poly (A) tail in the 3′-UTR. The TmCD63 ORF encoded a protein of 235 amino acids with a predicted molecular weight of 26.3 kDa. The encoded protein had an isoelectric point of 5.885 and a charge of −3.675 at pH 7.0 as predicted by LASERGENE software. The 235 amino acids were categorized into 17 strongly basic, 21 strongly acidic, 94 hydrophobic and 65 polar amino acids. The extinction coefficient of the protein was predicted to be 29,755 M^−1^·cm^−1^, assuming all pairs of Cys residues form cystines and 28,880 M^−1^·cm^−1^, assuming all Cys residues are reduced. The computed instability index was 33.08, which predict the protein to be stable with an aliphatic index of 98.64. The Grand Average of Hydropathy (GRAVY) was calculated as the sum of hydropathy values of all amino acids in the sequence and computed to be high at 0.469. The signal prediction software did not show any signal peptide sequence for the protein. Asparagines at position 119 (*N*-glycosylation potential of 0.5774) and at position 159 (*N*-glycosylation potential of 0.6819) were predicted to be available for glycosylation with a threonine at the 3rd position of the consensus tripeptides.

**Figure 2:**
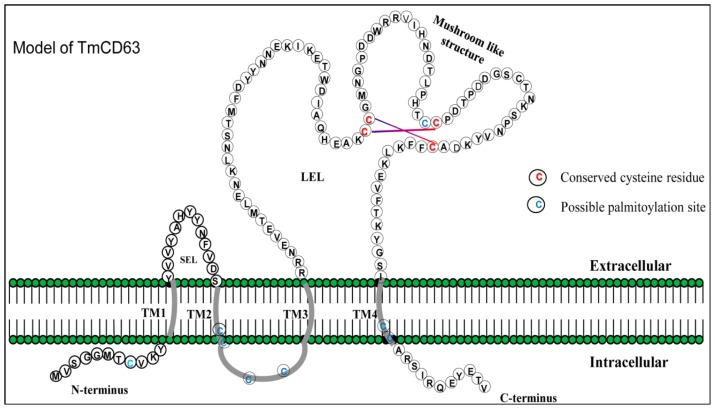
Schematic illustration of the structural design of TmCD63. The short extracellular loop (SEL), large extracellular loop (LEL) and the intracellular *N*- and *C*-terminal fragments are depicted with single-letter amino acid code. The transmembrane regions and the intracellular loop region are highlighted in grey. The possible juxtamembrane palmitoylated cysteines are indicated in blue. The conserved cysteines, which might form a functional mushroom architecture by disulphide bonds within the highly conserved “Cys-Cys-Gly” motif in the LEL, are indicated in red.

TMHMM analysis of the deduced amino acid sequence of TmCD63 indicated that the protein has four transmembrane domains (TM) (Figure S1) and can be categorized into TM_4_SF members. Apart from the four conserved TM domains spanning 23 amino acids, the protein consists of short *N*- and *C*-termini, short extracellular loop (SEL) and highly variable large extracellular loop (LEL). The TM1 domain begin from the 12th residue to the 34th residue, the TM2 domain from the 49th residue up to the 71st residue, the TM3 domain from 83rd amino acid residue to the 105th residue and the TM4 domain spanned from the 202nd to the 224th residue. The short *N*- and *C*-terminus of the protein spanned 11 amino acid residues each, whereas the SEL contained 13 amino acid residues. The LEL of the protein covered 97 amino acid residues and contained the highly conserved “Cys-Cys-Gly” motif, as well as a highly conserved “Cys188”, which defines the tetraspanin family members. The cysteines in the “Cys-Cys-Gly” motif formed a mushroom-like structure due to the presence of two intricate disulphide bonds. The “Pro-X-Ser-Cys” and “Cys-Cys-Gly” motif found in the LEL are hallmarks of the tetraspanin family (Figure 2). The model structure based on the amino acid sequence of TmCD63 also shows the presence of 8 palmitoylation sites, mostly with the juxtamembrane cysteine residues (Table 1). The short *N*-termini contained a palmitoylated cysteine at position 8, TM2 at position 71, intracellular sequence at positions 72 and 75 and TM4 at positions 222 and 223, excluding the SEL cysteine at position 166 that seems to be highly conserved as well. This is excluding the highly conserved cysteine residue in the SEL at position 188 (13 amino acid residues before TM4). The Kyte-Doolittle hydropathy plot of TmCD63 (Figure S2) revealed the four TM domains characteristic of the superfamily, as well as short extracellular (EC) region 1 and long EC2. The TM domains clearly showed a higher hydropathy score compared with the extracellular regions.

### 2.2. Phylogenetic Analysis of TmCD63 and Secondary Structure Prediction

**Figure 3:**
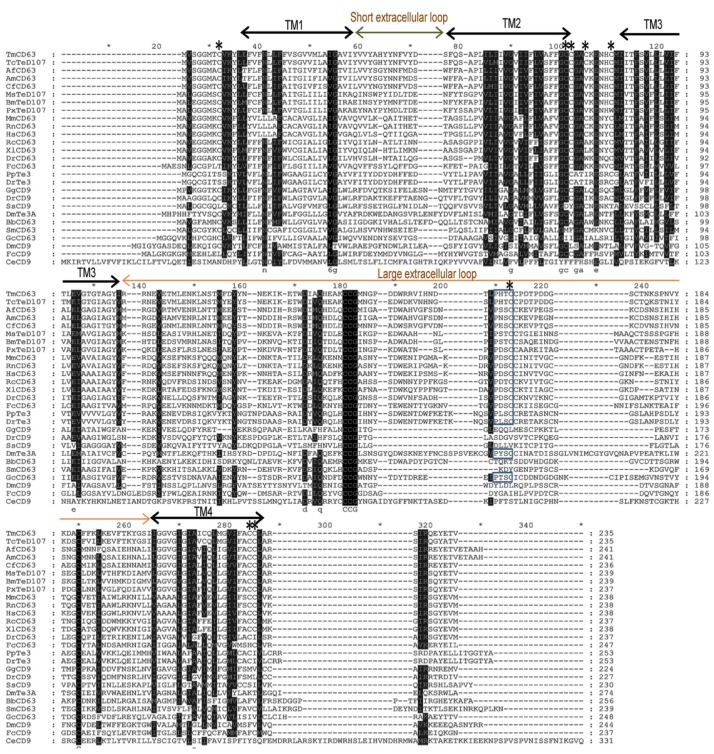
Sequence alignment of tetraspanin family members at the amino acid level. Tetraspanin sequences from various animal groups including insects were extracted using BLASTP program at NCBI. Alignment was carried out using ClustalX 1.83 program and further annotated by GeneDoc software. Identical residues of sequences including the highly conserved “Cys-Cys-Gly” motif are shown in black. The “Pro-X-Ser-Cys” motif is boxed. The transmembrane (TM) domains, short and large extracellular loops are indicated for the sequences. The predicted palmitoylation cysteine residues are indicated by asterisks.

Since tetraspanins have many family members, sequences of model animals from invertebrate and vertebrate groups based on combination of BLAST and the cysteine patterns of LEL of tetraspanins were selected for analysis; (Figure 3). The alignment shows that the TM regions (I–IV) are more conserved than the LEL region. The “Cys-Cys-Gly” motif and “Cys188” show high degree of conservation in the LEL of the tetraspanin family members. Additionally, the palmitoylated cysteines show greater degree of conservation among the sequences included in the alignment. The “Pro-X-Ser-Cys” motif was found in *T. molitor* as well as its close relative *Tribolium castaneum* tetraspanin D107 (TcTeD107), although it was absent in the *Branchiostoma* and *Schistosoma* CD63 apart from the CD9 family of the tetraspanins.

**Figure 4:**
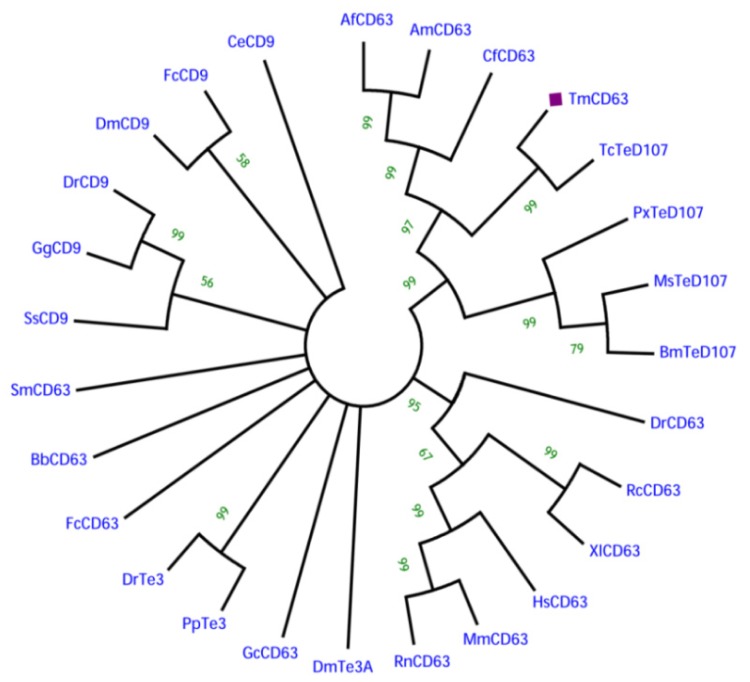
Phylogenetic analysis based on tetraspanin amino acid sequences from various animals including insects. The phylogenetic tree was constructed using the neighbor-joining algorithm method and bootstrapped 1000 times. The analysis was performed using the MEGA (version 5.0) program. The evolutionary distances were computed using the Poisson correction method and are in the units of the number of amino acid substitutions per site. The abbreviations and accession numbers of the tetraspanin amino acid sequences were as follows: *Tribolium castaneum* TeD107 (TcTeD107; XP_971111.1), *Camponotus floridanus* CD63 (CfCD63; EFN70056.1), *Apis florea* CD63 (AfCD63; XP_003695432.1), *Apis mellifera* CD63 (AmCD63; XM_625096.2), *Manduca sexta* TeD107 (MsCD63; AAF90147.1), *Bombyx mori* TeD107 (BmTeD107; NP_001040319.1), *Plutella xylostella* TeD107 (PxTeD107; ABI89030.1), *Rattus norvegicus* CD63 (RnCD63; NP_058821.1), *Mus musculus* CD63 (MmCD63; NP_031679.1), *Rana catesbeiana* CD63 (RcCD63; ACO51866.1), *Xenopus laevis* CD63 (XlCD63; Q7SY95), *Danio rerio* CD63 (DrCD63; Q6P2T9), *Homo sapiens* CD63 (HsCD63; P08962), *Fenneropenaeus chinensis* CD63 (FcCD63; EU095883), *Fenneropenaeus chinensis* CD9 (FcCD9; EF032649), *Drosophila melanogaster* tetraspanin 3A (DmTe3A; AAF90139), *Drosophila melanogaster* CD9 (DmCD9; CAA17687), *Branchiostoma belcheri* CD63 (BbCD63; Q6W8Q0), *Schistosoma mansoni* CD63 (SmCD63; Q8ITD7), *Pongo pygmaeus* tetraspanin 3 (PpTe3; Q5RE11), *Danio rerio* tetraspanin 3 (DrTe3; Q4V915), *Gallus gallus* CD9 (GgCD9; Q9IBC9), *Danio rerio* CD9 (DrCD9; Q7ZUH9), *Salmo salar* CD9 (SsCD9; Q8AYJ0), *Caenorhabditis elegans* CD9 (CeCD9; CAA99805) and CD63 of *Genodia cydonium* (GcCD63; CAA70025).

**Figure 5:**
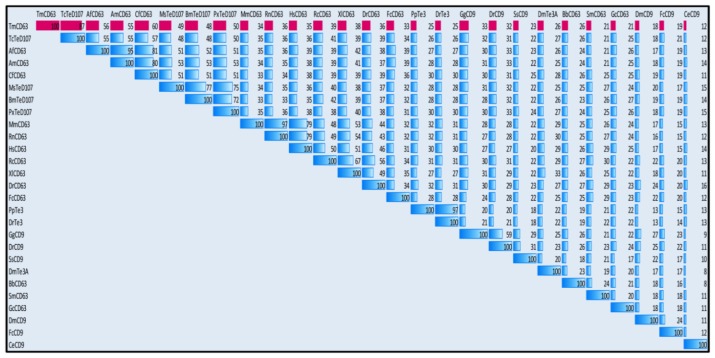
Percentage pairwise identities of TmCD63 with tetraspanin amino acid sequences from various animals including insects. The percentage identities have been highlighted using blue data bars with red bars indicating identities of TmCD63.

In order to understand the phylogenetic relationship of TmCD63 with other tetraspanin family members from invertebrates and vertebrates, an assessment was made based on the amino acid sequence of similar proteins (Figure 4). Three significant clusters were noticed comprising CD63s from vertebrates, a completely independent CD9 cluster and insect tetraspanin cluster. The insect tetraspanin cluster was found to delineate into two identifiable clades belonging to the CD63 and tetraspanin D107. TmCD63 was found to group within the insect tetraspanin cluster and was expectedly close to its relative *T. castaneum* tetraspanin (TcTeD107). This is the first reported CD63 from the coleopterans and therefore it was not possible to have other CD63 sequences from coleopterans in the analysis. It will be interesting to note the evolutionary position of TmCD63 with its orthologs as more of their members are identified and reported. The coleopteran CD63 group that branched separately had a better sequence identity with CD63 clade compared with tetraspanin D107 clade. The vertebrate CD63 family clustered separately from the insect cluster with the amphibian CD63 (RcCD63 and XlCD63) and mammalian CD63 (MmCD63 and RnCD63) showing a better bootstrap value. The CD9 family members formed a separate group and found to less closely relate with the insect tetraspanin and mammalian CD63 cluster. The results of the phylogenetics study were also evaluated with a percentage identity matrix plot (Figure 5). As revealed before, there exists a close identity of 87% with the tetraspanin of *T. castaneum*, followed with 50%–56% identity with other insect tetraspanin family members with the maximum identity of 60% with *Camponotus floridanus* CD63 (CfCD63). A sequence identity of 33%–39% was noted with the vertebrate CD63 family with the maximum identity of 39% with *Xenopus laevis* CD63 (XlCD63). A lesser identity of 12% to 33% was noticed with tetraspanin 3 and CD9 family members.

**Figure 6:**
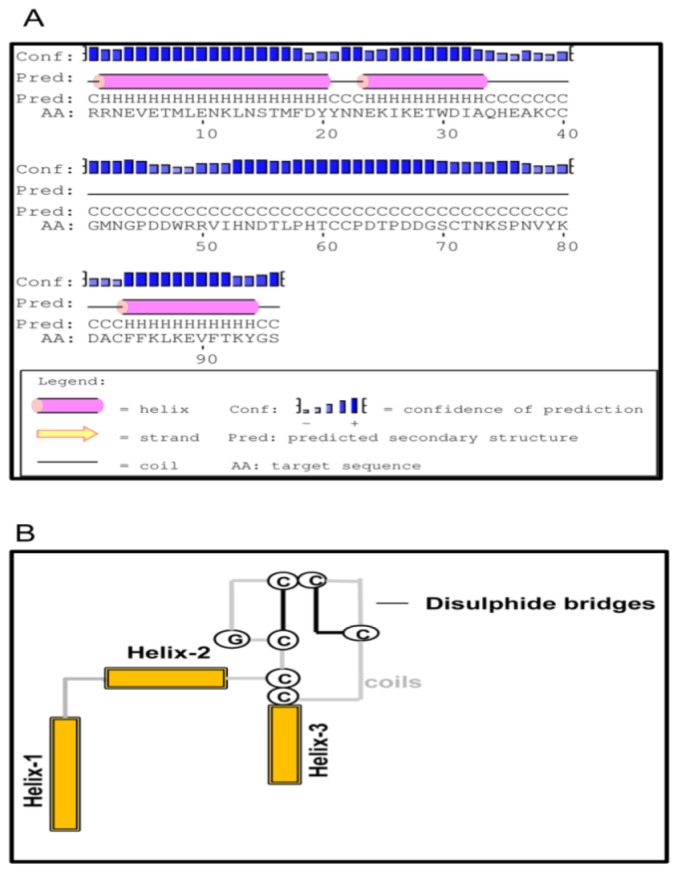
Modeling of LEL topology of TmCD63. (**A**) Secondary structure prediction of TmCD63 LEL using PSI-PRED software. The helical regions (three in number) are demarcated by an extensive coil region that encompasses the mushroom-like structure, characteristic of tetraspanin superfamily; and (**B**) Schematic representation of TmCD63 LEL topology containing six cysteine residues.

The LEL of CD63 family of proteins was known to be highly variable in their LEL, with maximum identity seen within the conserved TM domains. This led us to further investigate LEL of TmCD63 by means of *in silico* structure prediction methods. The LEL of TmCD63 was found to comprise of a core formed by three helices a, b and e, and this core structure is conserved among the tetraspanins (Figure 6A). Also, the 6 cysteine residues in the LEL suggests that TmCD63 would belong to group 6a, wherein the three helices are closely spaced following up with the coil region that can format itself to a mushroom-like structure with two disulfide bridges (Figure 6B). The prediction was not conclusive towards the helices c and d that spans the variable portion of LEL.

### 2.3. Developmental and Tissue Distribution of TmCD63

**Figure 7:**
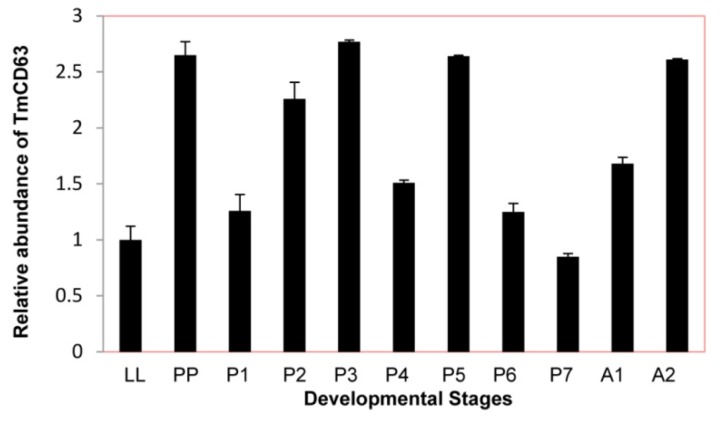
Developmental expression patterns of TmCD63 in mealworm beetle *T. molitor*. Ribosomal protein L27a (*T. molitor*) was used as an internal control to normalize RNA levels between samples. The relative expression was calculated by comparing the cycle threshold value of TmCD63 to L27a. Vertical bars represent standard deviations (*n* = 3). The analysis was performed using real-time PCR based on SYBR green. LL: Late larva; PP: Prepupa; P1: Pupa day 1; P2: Pupa day 2; P3: Pupa day 3; P4: Pupa day 4; P5: Pupa day 5; P6: Pupa day 6; P7: Pupa day 7; A1: Adult day 1; A2: Adult day 2.

In an attempt to study the basal expression level of novel CD63 homologue in different developmental stages of *T. molitor*, late larval, pupal and adult stages were chosen for the study (Figure 7). Although their seems to be little difference in the constitutive expression of TmCD63 in different developmental stages, the CD63 levels were found to be slightly higher at pre-pupal and early days of the pupal stage with a marginal decline towards the last days of the pupal stage.

**Figure 8:**
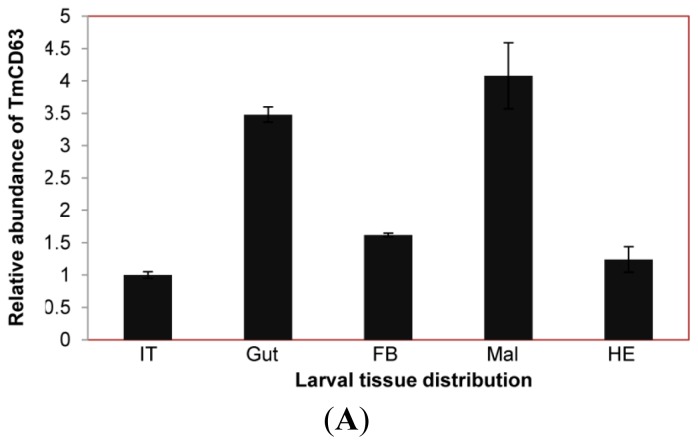

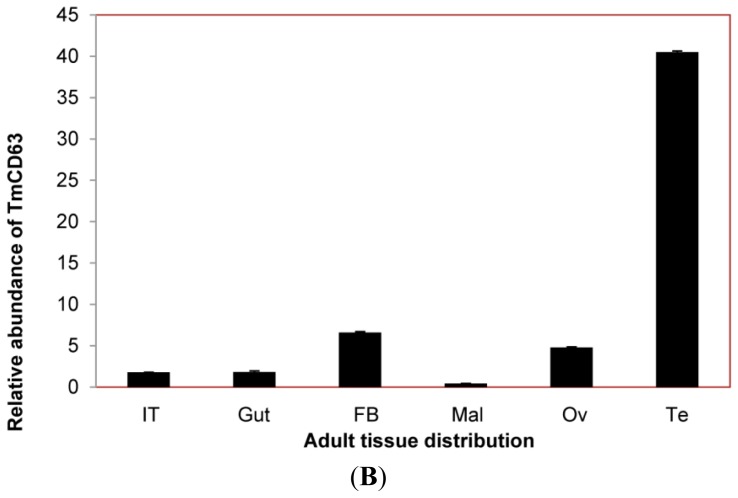
Tissue-specific expression study of TmCD63 in mealworm beetle *T. molitor*. (**A**) The distribution of the transcript in last instar larval tissues; and (**B**) The distribution of the transcript in 2-day-old adult tissues. Ribosomal protein L27a (*T. molitor*) was used as an internal control to normalize RNA levels between samples. The relative expression was calculated by comparing the cycle threshold value of TmCD63 to L27a. Vertical bars represent standard deviations (*n* = 3). The analysis was performed using real-time PCR based on SYBR green. IT: integument; Gut: fore-, mid and hindgut; FB: Fat body; Mal: Malphigian tubules; He: Hemolymph; OV: Ovary; Te: Testis.

The expression of TmCD63 mRNA was investigated in various tissues of final instar larvae and adult of *T. molitor* (Figure 8). TmCD63 was highly expressed in the gut and Malphigian tubules of the larvae, but detected at low levels in fat body, integument and hemocytes. The relative expression level of TmCD63 was the highest in Malphigian tubules among the five tissues with about 4-fold higher presence as compared with the integument. The TmCD63 mRNA was found to be strikingly higher in the adult testis with ~40 fold expression compared with the reference of Malphigian tubules. We therefore speculate that TmCD63 plays a key role in differentiation of the testicular cells in adult of *T. molitor.* The high expression of TmCD63 transcripts in the potent immune organs of *T. molitor* larvae may suggest its role in innate immune mechanisms of insects. Expression levels of *L27a* gene of *T. molitor*, employed here as an internal control, appeared to be similar and unchanged among developmental stages.

### 2.4. Time Course Analysis of TmCD63 after Immune Elicitor Challenge

In an attempt to study the putative role of the novel TmCD63 in innate immune mechanisms of *T. molitor*, we conducted a time-course study (for 24 h) in the whole larvae after challenge with Mycoplasma (*Acholeplasma laidlawii* lysate and live cell), intracellular Gram-positive bacterial pathogen *Listeria monocytogenes*, fungal cell wall component (β-1,3 glucan) and bacterial cell wall components [Lys- and DAP-type peptidoglycans (PGNs)]. The gene expression levels were quantified relative to the expression levels found in the uninjected larvae.

**Figure 9:**
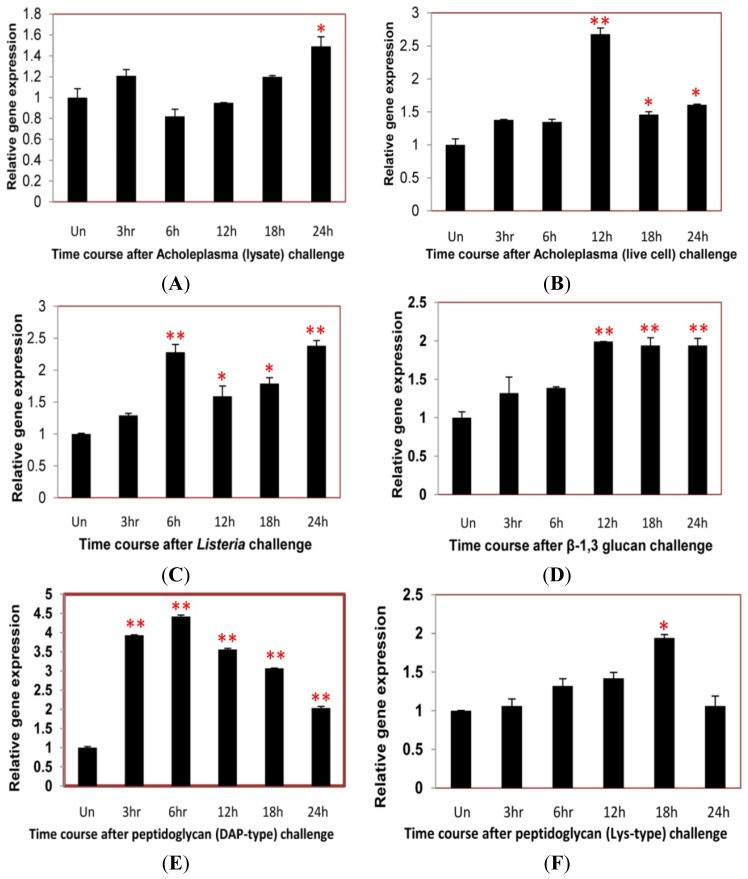
Time-course expression of CD63 homologue in *T. molitor* after injection with (**A**) *Acholeplasma laidlawii* lysate; (**B**) *Acholeplasma* live cell; (**C**) *Listeria monocytogenes*; (**D**) β-1, 3 glucan; (**E**) DAP-type Peptidoglycan and (**F**) Lysine-type (Lys-type) peptidoglycan. Total RNA was extracted from *Tenebrio molitor* larvae after interval of 3, 6, 12, 18 and 24 h post inoculation and profiled by real-time PCR. Ribosomal protein L27a (*Tenebrio molitor*) gene was used as internal control. Vertical bars represent standard deviations (*n* = 3). * p < 0.05, ** p < 0.001.

After *Acholeplasma* lysate stimulation, there was no striking upregulation of TmCD63 until about 24 h of challenge (Figure 9A). Similarly, with *Acholeplasma* live cell injection, the expression level didn’t show a significant change, except after 12 h interval, where the expression levels was maximum (Figure 9B). Challenge with a serious intracellular pathogen, such as *L. monocytogenes* led to a significant upsurge in the expression levels of the gene at 6 h post-stimulation and was maintained till 24 h (Figure 9C). Similar trend was also observed after injection of β-1,3 glucan where the expression was significant at 12 h post-stimulation and maintained until 24 h (Figure 9D). TmCD63 mRNA was significantly upregulated by stimulation with DAP-type PGN, with almost 4–5 fold induction during the first 6 h of infection. The upregulation of TmCD63 transcripts were 2–4 fold at after 6 h post-injection (Figure 9E). A more significant expression of TmCD63 transcript was found at 18 h post-stimulation of Lys-type PGN (Figure 9F).

## 3. Discussion

Tetraspanins are an evolutionarily conserved family of proteins that have been investigated for their potential functions in regulating cell morphology, motility, invasion, fusion and signaling as organizers of multi-molecular membrane complexes and have been found to be expressed in a wide variety of organisms, encompassing invertebrates and vertebrates [7,42]. The exhaustive collation of information on the existence of tetraspanin-family members has been possible due to the recent whole-genome sequencing of various organisms, and this includes 37 family members identified from *Drosophila melanogaster* [4]. The EST information generated from our earlier work on the coleopteran beetle, *Tenebrio molitor* [41], identified a clone that was evaluated to be a homologue of CD63 and is an important candidate for the study of innate immunity in the insect. Molecular cloning and subsequent *in silico* analysis of TmCD63 ORF and protein was critical in assessing its sequence characteristics and validating their affinity with other tetraspanin-family members.

The deduced amino acid sequence of TmCD63 was modeled into four TMs (TM I–IV), short *N*- and *C*-termini, SEL and the LEL containing the “Cys-Cys-Gly” conserved motif, as well as a conserved “Cys188” as is known from other tetraspanin family-members (TM_4_SF). It is known that the proteins in this family contain 200–300 amino acid residues that include four TMs, one SEL region containing 13–30 amino acids, a short intracellular sequence, and a variable LEL containing the “Cys-Cys-Gly”, crucial in the determination of functional specificity [43]. The LEL domain of TmCD63 protein was topologically located between TM3 and TM4 and contained six invariant cysteine residues, although a variation of four to eight cysteine residues allowing the formation of two to four disulphide bridges, have been reported [43]. It is also interesting to note that the last cysteine (Cys188) in the LEL of TmCD63 is in proximity to helix-3 and TM4 and seems to be completely conserved within the tetraspanin superfamily, suggesting an important conserved role for this residue. In support of this, the 3D structure of CD81, a representative tetraspanin model, defines the LEL into two domains, one conserved and one variable [44]. The helices a, b and e in LEL seem to be more conserved, whereas helices c and d that traverse the mushroom-like projection in the tetraspanins are considered to be the most evolutionarily divergent sequences; as is the case of CD81 molecules from different species [45,46]. TmCD63 LEL seems to fit into group 6a (CCG--CC---C--C), out of the six different amino acid motifs (4a, 6a–c and 8a,b) that have been observed within the tetraspanin superfamily. Out of the six cysteine residues, four of them are bound by two disulfide bridges in TmCD63. Disulfide bridges are responsible in providing a structural scaffold that enables tolerance of wide-variability in the inter-cysteine loops, probably enabling adaptation to diverse protein interactions.

Protein palmitoylation, considered as a modification of juxtamembrane cysteine residues, plays a crucial role in the tetraspanin web due to the formation of thioester-linkages in the protein. All tetraspanin proteins (including CD9, CD63, CD81, CD82, CD151, *etc.*) seem to be palmitoylated, as also found in the case of TmCD63 where eight palmitoylated cysteines have been identified. Earlier reports involving site-directed mutagenesis have suggested the utilization of intracellular membrane proximal cysteines (most specifically the cysteines in short *N*- and *C*-termini) for palmitoylation [47]. This being conserved in most other tetraspanin family members including TmCD63 (cysteine at positions 8, 222 and 223), can be considered as the most important residues for palmitoylation; although other cysteines proximal to short intracellular loop may be involved as well. The functional relevance of such modifications is in improving the networking and interactions of tetraspanins with other tetraspanins or other proteins, through stabilization of the “tetraspanin web” [48]. Association of such tetraspanin-enriched microdomains (TEMs), especially the lysosomal tetraspanin CD63, in the intracellular trafficking of type I membrane protein synaptotagmins from Golgi complex to lysosomes and efficient phagocytosis in macrophages have been demonstrated to be palmitoylation-dependent [49]. Additionally, the presence of an YXXØ [where tyrosine (Y) is succeeded by any two amino acids denoted as “X” and Ø representing a bulky hydrophobic residue] motif in the *C*-terminal region is found critical in cell sorting, including lysosomal and basolateral targeting, although the mechanism is not clear [50]. TmCD63 does contain the tyrosine-based motif as YETV residues in which valine represents a considerably bulky residue with free energy transfer value of 1.70 KCal. The alignment configured for the present study reported the conspicuous presence of the motif in mostly all insect tetraspanin family members, albeit in mammals valine residue was found replaced with methionine. Mammalian CD63, with a *C*-terminal YEVM sequence interacts with a PDZ domain in a transmembrane and connector protein syntenin-1 [19]. The *N*-glycosylation sites of TmCD63 and other TM proteins may serve as a function for signal transduction, intercellular stability and subunit folding [51].

TmCD63 full-length amino acid sequence was further scrutinized by conducting a multiple sequence alignment (MSA) and subsequently the phylogenetic analysis. As expected, the protein showed highest identity with tetraspanin D107 from its close relative, the red flour beetle, *T. castaneum*. The phylogenetic tree showed that the beetle tetraspanins formed a separate branch within the insect tetraspanin cluster. The molecular evolution of the superfamily involves rapid amino acid divergence and considerable changes in length in the LEL. The scope of phylogenetic analysis was exhaustive in the present study, to delineate the evolutionary position of TmCD63. Phylogenetic classifications of tetraspanins in a broader set of eukaryotic organisms have been reported [52,53]. One important analysis focused on the “Cys-Cys-Gly” motif of the LEL loop that seems to be absent in tetraspanins from Choanoflagellates, Tsp11 class of fungal tetraspanins *Phytopthora*, plants as *Selaginella*, *Oryza* and *Arabidopsis* [54]. The “Cys-Cys-Gly” motif is a characteristic feature in almost every group of fungi and animals and also in protists like unikonts that are more closely related to them. This suggests that the lack of “Cys-Cys-Gly” motif may be an ancestral characteristic of plants and organisms related to plants [6]. Another pattern that was mapped onto the phylogenetic tree was the cysteine patterns that seem to cluster together with strongly supported clades in the tetraspanin tree [6]. In this scenario, cysteine pattern 8a is characteristic of TSPAN15L, 6a of CD63L and 6c of the TSPAN13L group of tetraspanins. Insect tetraspanins have been divided into four groups: CD63-like, CD151, TSPAN5, TSPAN7 and TSPAN31, with three families showing high divergence from other insect and non-insect tetraspanins, suggesting specialized roles for these. CD63 is likely to have a more ancient origin as it is associated with a gene expansion in *Drosophila* and also has been reported in sponges [55].

The great biological relevance of tetraspanin family members in terms of varied functionalities prompted us to investigate the spatial and tissue-specific distribution of the CD63 homologue in the model insect, *T. molitor*. The expression level of the gene was found to be homogenous in the developmental stages with slightly higher levels in early pupal and adult stages. The TmCD63 mRNA was found constitutively expressed among the larval tissues and was significantly higher in the immune organs such as gut and Malphigian tubules. Most of expression in the adult tissues was observed in the germ cells, especially testis, suggesting its role in germ cell differentiation and maturation. The wide distribution of TmCD63 in immune-related tissues of the larvae led us to further investigate the role of the gene in innate immune response against certain widely used immune elicitors such as the cell wall components of the fungi (β-1,3 glucan), Gram-positive bacteria (Lys-type PGN) and Gram-negative bacteria (DAP-type PGN). The expression of TmCD63 showed an upregulation, more significantly, in the late hours of challenge in case of β-1,3 glucan and Lys-type PGN. Most strikingly, DAP-type PGN showed a significant induction of TmCD63 transcripts within 3 h of injection and was maintained at later stages after challenge. The maintenance of high mRNA levels in the later phases after challenge might be very important in the development of pathological symptoms from an intracellular pathogen, *L. monocytogenes*. Earlier report has documented the intracellular expression of CD63 by fluorescence microscopy and suggested that upon infection with the pathogen, endogenous CD63, as well as, CD9 and CD81 were recruited to the bacterial entry site, though finally only CD81 was required for bacterial internalization, identifying for the first time the role of tetraspanins in *L. monocytogenes* entry into target cells [56]. RNA interference data with CD81 have suggested the membrane organizer action required for the integrity of signaling events at *L. monocytogenes* entry sites. CD81 also acts as receptor for hepatitis C virus, and neutralizes anti-HCV antibodies, thus inhibiting the binding of virus to LEL of CD81 [57]. Other reports of recruitment of CD82 to phagosomes in response to pathogenic fungi as *Cryptococcus neoformans*, *Candida albicans* and *Aspergillus fumigatus* and bacteria as *E. coli* and *Staphylococcus aureus* seems interesting [58]. In addition, it has been demonstrated that CD63 recruitment to *C. neoformans* phagosomes is dependent on phagosomal acidification [59]. The roles of CD63 with other tetraspanins such as CD9 and CD151 in preventing adherence of *Neisseria meningitides*, *Staphylococcus aureus*, *Neisseria lactamica*, *E. coli* and *Streptococcus pneumoniae* to human epithelial cells have been observed with the help of tetraspanin antibodies generated against LEL and small interfering RNAs (siRNAs) [60]. Additionally, development of exciting new therapeutic drugs/vaccines by using the recombinant form of tetraspanins, especially the LEL has already been reported [61,62]. These observations support our hypothesis for TmCD63 that it can be implicated as facilitating and improving the immune status of the insect by recruitment and rehabilitation at phagosomes, specifically as the transcript gets expressed towards the later stage of infection.

## 4. Experimental Section

### 4.1. Insect Rearing

Larvae of mealworm beetle, *T. molitor* procured from College of Pharmacy, Pusan National University, Busan, Korea, were reared on wheat bran meal in an environmental chamber at 25 ± 1 °C with 60% ± 5% relative humidity and a 16:8 h light and dark cycle. Only last instar larvae were used for all experiments unless otherwise stated. Tissues such as the gut, Malphigian tubules, fat body, integument, and hemocytes were collected and pooled (*n* = 3) from larvae and adults (in this case including ovary and testis) for expression analysis. The tissues were snap-frozen in liquid nitrogen, and stored at −80 °C until RNA extraction.

### 4.2. Chemicals and Strains

All chemicals used for the experiments were of analytical grade, obtained from Sigma Chemical Co. (St. Louis, MO, USA) until otherwise mentioned in the text. The Gram-positive strain, *L. monocytogenes* (American type culture collection—ATCC 7644) and *A. laidlawii* (*Mycoplasma*) strain (Korean Collection of Type Cultures—KCTC 1621) used for immune elicitor challenge studies were procured from Pusan National University, Busan, Korea.

### 4.3. Construction of cDNA Library of T. molitor Larvae

The total RNA from *T. molitor* larvae was isolated by TRIzol reagent (Molecular Research Centre, Inc. Cincinnati, OH, USA) after homogenization using TissueLyser (Qiagen, Valencia, CA, USA) and subsequently mRNA was purified using Absolutely mRNA Purification Kit (Stratagene, Santa Clara, CA, USA). The cDNA library was synthesized using Express cDNA Synthesis Kit (Stratagene). The cDNAs of more than 500 bp in length were ligated into pBK-CMV vector and packaged using the ZAP expression cDNA Gigapack^®^ III Gold cloning kit (Stratagene) according to manufacturer’s instructions. Three clones corresponding to the partial fragment of CD63 homologue (TmCD63) with 5′ untranslated region (UTR), were identified by conducting BLASTx and Swissprot analysis (EMBL-EBI, Hinxton, Cambridge, UK).

### 4.4. Cloning of the Full-Length cDNA of TmCD63

One of the clones (Nor-*Tenebrio*-contr-5-4a-PE_P10) having the longest insert size was used as a template to design the 3′ rapid amplification of cDNA ends (RACE) PCR primers (Table S1). The 3′ end of TmCD63 was cloned using SMARTer™ RACE cDNA amplification kit (Clontech laboratories, Inc. Mountain View, CA, USA) according to manufacturer’s instructions. One microgram of total RNA and 1 μmol·L^−1^ of each primer were used to synthesize the 3′-RACE-ready cDNA with an oligo-dT adaptor primer. Because of the terminal transferase activity of the SMARTScribe reverse transcriptase used, the first strand cDNAs possess the adaptor primer sequence with 3′ end. For 3′-RACE, the first PCR was carried out with the universal primer and gene specific forward primer 1 (3′-GSP1), followed by nested gene-specific forward primer 2 (3′-nGSP2). The PCR amplification was done as follows: denaturation at 94 °C for 3 min, annealing at 58 °C for 30 s, extension at 72 °C for 1 min for 25 cycles. The nested PCR products were extracted and separated in 1% agarose gel by using AccuPrep PCR and Gel purification kit (Bioneer Company, Daejon, Korea) and subsequently cloned into TOPO TA cloning vector (Invitrogen Corporation, Carlsbad, CA, USA) and subsequently transformed into competent *E. coli* DH5α cells and sequenced. Specific primers and nested primers for amplification have been listed in Table 1. The full-length cDNA sequence of TmCD63 has been submitted to European Nucleotide Archive-European Molecular Biology Laboratory (ENA-EMBL, Hinxton, Cambridge, UK) with an accession number HG316497.

### 4.5. TmCD63 Sequence and Phylogenetic Analysis

TmCD63 cDNA sequence was analyzed by UltraEdit-32 Professional Text/HEX editor (version 12.00, IDM Computer Solutions Inc., Hamilton, OH, USA) software package and deduced amino acid sequence was predicted by Open Reading Frame finder at NCBI [63]. The sequences of CD63 from other representative insects, as well as, the mammalian tetraspanins were obtained from GenBank, and alignments were conducted using Clustal X (version 2.0.12, University of Strasbourg, Strasbourg, France) [64]. Subsequently, phylogenetic analysis of the full length amino acid sequence was conducted using MEGA version 5.0 software (The Biodesign Institute, Tempe, AZ, USA) [65]. A phylogenetic tree was constructed using the neighbor joining method [66]. To evaluate the branch strength of the phylogenetic tree, bootstrap values representing 1000 replicates were taken for analysis. Percentage identities of the full length amino acid sequence of TmCD63 and other representative species were calculated using ClustalW2 (EMBL-European Bioinformatics Institute, Cambridge, UK).

The transmembrane domains in proteins were predicted by TMHMM (Technical University of Denmark, Lyngby, Denmark). The palmitoylation sites at high threshold were predicted by CSS-Palm 3.0 (Cuckoo workgroup, Wuhan, China) [67]. The prediction of the putative signal peptide sequence was done at the Signal 4.0 server [68]. The ORF base composition and protein statistics including the theoretical MW and isoelectric point (pI) were performed using the EditSeq and Protean tool of Lasergene 9.0 software (DNASTAR Inc., Madison, WI, USA). The prediction of *N*-glycosylation sites was confirmed at NetNGlyc 1.0 server (Technical University of Denmark, Lyngby, Denmark). ProtScale [69] at ExPASy bioinformatics resource portal was used to predict the hydrophobicity quality of the protein [70]. ProtParam [71] at ExPASy bioinformatics resource portal was used for computing various physical and chemical parameters [72].

### 4.6. Tissue Distribution and Developmental Expression Profile of TmCD63 by Quantitative Real-Time PCR

Total RNA was extracted from different developmental stages of *T. molitor* metamorphosis such as last instar larvae, pre-pupae, pupae (days 1 to 7) and adults (days 1 and 2). In addition, various tissues from gut, Malphigian tubules, fat body, integument as well as ovaries and testis from *T. molitor* were dissected and collected to isolate total RNA by using SV Total RNA isolation kit (Promega Corporation, Madison, WI, USA) following manufacturer’s protocol. For hemocytes collection, hemolymph were collected from abdomen using an equal volume of modified Alsevier’s solution (MAS) as anticoagulant [73] and centrifuged for 10 min at 800 × *g*, 4 °C. RNA concentration and purity of each sample were quantified in NanoDrop 2000 UV-Vis Spectrophotometer (Thermo Scientific, Wilmington, OH, USA; A260/A280 nm ratios >1.8). The RNA integrity was checked by separation on ethidium bromide stained 1% agarose gels. A sample of 2 μg of total RNA was reverse-transcribed using Oligo (dT)_18_ primer (Invitrogen, Carlsbad, CA, USA) at 72 °C for 5 min. Quantitative real-time PCR was performed on Exicycler™ 96 real-time quantitative thermal block (Bioneer, Korea) using primers (Table S1) at an initial denaturation of 94 °C for 3 min, followed by 28 cycles at 94 °C for 40 s, 72 °C for 90 s and 72 °C for 10 min. Each treatment was independently replicated three times. The 2^−ΔΔ^*^C^*^_t_^ method (where ΔΔ*C*_t_ = Δ*C*_t target_ − Δ*C*_t reference_) was employed to analyze the expression levels of TmCD63 [74]. The gene expression levels were normalized to ribosomal protein L27a (accession number X99204), which served as an internal control. The data were presented as relative mRNA expression levels [mean ± standard deviation (SD), *n* = 3].

### 4.7. Immune Elicitors and Challenge Studies

Immune elicitors as the intracellular pathogen, *L. monocytogenes*, *A. laidlawii* lysate and live cells, β-1,3 glucan, Lys- and DAP-type peptidoglycan were procured from College of Pharmacy, Pusan National University, Korea. Time course of TmCD63 induction was studied in the whole larvae by injection of 4 μL of immune elicitors into the last instar larvae of mealworm beetle, *T. molitor* with the Picospritzer III (Parker Hannifin, Hollis, NH, USA). The control group was injected only with phosphate buffer saline (PBS) (0.14 M sodium chloride, 3 mM potassium chloride, 8 mM disodium hydrogen phosphate dodecahydrate, 1.5 mM potassium phosphate monobasic, pH 7.4), as the wounding buffer. Total RNA from the larvae was collected after 3, 6, 12, 18 and 24 h post-injection. All the samples were frozen in liquid nitrogen and then stored at −70 °C until use.

### 4.8. Time-Course Analysis of TmCD63 by Quantitative Real-Time PCR

Total RNA from *T. molitor* was prepared using Total RNA Isolation kit (Promega Corp., Madison, WI, USA) according to the manufacturer’s protocol. Two micrograms of total RNA was reverse-transcribed in a 50 μL reaction mixture with a High capacity cDNA Reverse Transcription Kit (Bioneer, Korea). qRT-PCR was performed on Exicycler™ 96 Real-Time Quantitative Thermal Block (Bioneer, Korea). The 50-μL mixture including 1 μL of cDNA, 10 pmol of each primer (Table S1) and 25 μL of 1× LightCycler 480 SYBR Green (Takara Bio Inc. Shiga, Japan) was placed in 96 well plates. The PCR program was set with an initial denaturation of 95 °C for 20 s, followed by 40 cycles at 95 °C for 15 s, 60 °C for 1 min and 72 °C for 10 s.

The primers were designed using Primer Quest (Integrated DNA Technologies, Coralville, IA, USA) [75]. Each treatment was independently replicated three times. The 2^−ΔΔ^*^C^*^_t_^ method was employed to analyze the expression levels of TmCD63 and the value obtained denoted the *n*-fold difference related to the calibrator (uninjected samples). The data were presented as relative mRNA expression levels (means ± S.D., *n* = 3). The data were subjected to a one-way analysis of variance (ANOVA). Significant differences between the treated group and the corresponding control group at each time point were indicated with one asterisk for *p < 0.05* and two asterisks for *p <* 0.01.

## 5. Conclusions

Our discovery that TmCD63 mRNA is upregulated in response to injection of bacterial and fungal cell wall components, suggests that it has a role in innate immune responses in insects. Additional studies involving the use of recombinant CD63 and RNA interference approaches will further our understanding of the functions of CD63. We have generated an RNAi knockout model for the gene and are currently studying the localization and fidelity of TmCD63 as a lysosomal marker and its role in autophagocytosis against an intracellular pathogen, *L. monocytogenes*. The response of the molecule against invasion and penetration of the baculoviruses, especially nucleopolyhedrovirus, is also a contentious subject for further study.

## Supplementary Information



## Figures and Tables

**Table 1:** Predicted palmitoylation sites (CSS-Palm 3.0 software suite) in CD63 homologue of *T. molitor*.

Position	Peptide	Score	Cutoff	Cluster
8	MVSGGMTCVKYLLFV	0.595	0.308	Cluster A
71	FLVAFFGCCGACKEN	1.694	0.497	Cluster B
72	LVAFFGCCGACKENH	2.278	0.497	Cluster B
75	FFGCCGACKENHCMI	0.852	0.497	Cluster B
80	GACKENHCMIITFSV	2.322	1.225	Cluster C
166	NDTLPHTCCPDTPDD	0.806	0.497	Cluster B
222	LMGVIFACCLARSIR	3.433	0.308	Cluster A
223	MGVIFACCLARSIRQ	3.481	0.497	Cluster B
