# Supplementary material for: Molecular Cloning, Sequence Characterization and Expression Analysis of a CD63 Homologue from the Coleopteran Beetle, Tenebrio molitor
Source: Int J Mol Sci. 2013 Oct 15;14(10):20744–67. doi: 10.3390/ijms141020744 (PMC3821641; doi:10.3390/ijms141020744)
Supplement: Supplementary file 1 [file ijms-14-20744-s001.pdf]

Supplementary Information

Figure S1. Transmembrane domain prediction of TmCD63. The protein belongs to TM_4SF, as four transmembrane domains have been noted, the long strings showing the LEL motif of the tetraspanin.

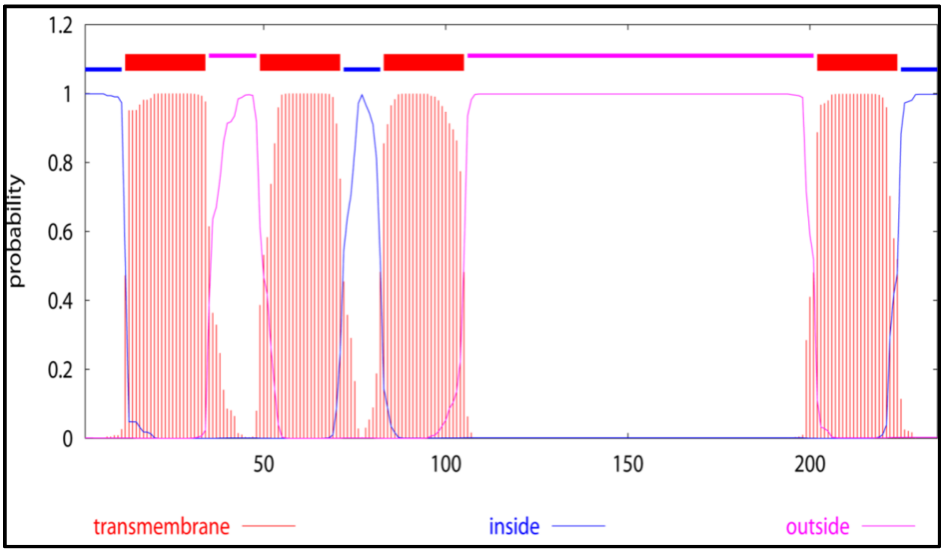

Figure S2. Kyte-Doolittle hydropathy plot of TmCD63. The hydropathic character of the protein was predicted using ProtScale at ExPASy bioinformatics resource portal. The four transmembrane domains of continuously high hydrophobicity are numbered, as are the short and long extracellular domains 1 and 2.

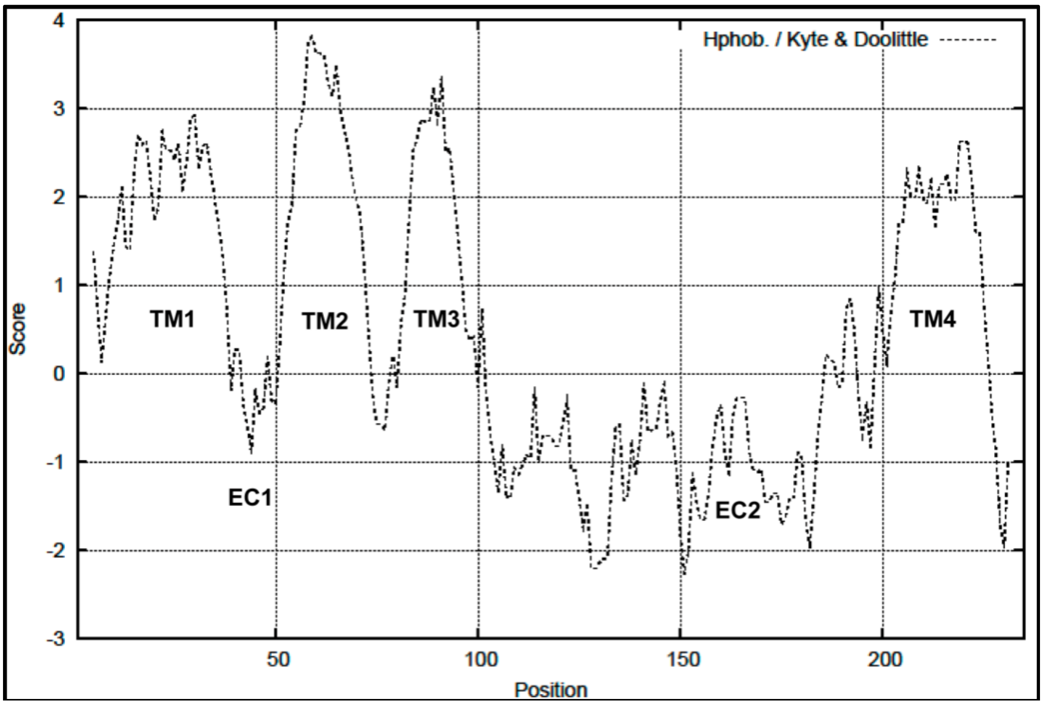

Table S1. Primers used in the present study.

Primers	Sequence (5'-3')
**RACE-PCR primers**	
3'-GSP1 (forward)	CTCTACCTCACACTTGCTGT
3'-nGSP2 (forward)	TTGGCATCGCGATTTGTCAGC
3'-CDS Primer A (OligodT adaptor)	AAGCAGTGGTATCAACGCAGAGTA$(T)_{30}$VN
SMARTer IIA oligonucleotide	AAGCAGTGGTATCAACGCAGAGTACXXXXX
Universal adaptor primer	CTAATACGACTCACTATAGGGCAAGCAGTGGTATCAACGCAGAGT
**Sequencing primers**	
M13-F (forward)	GTAAAACGACGGCCAG
M13-R (reverse)	CAGGAAACAGCTATGAC
**Real-time PCR primers**	
TmCD63 (forward)	TTGCTGTCCAGACACACCTG
TmCD63 (reverse)	CAGCTGACAAATCGCGATCG
L27A (forward)	TCATCCTGAAGGCAAAGCTCAGT
L27A (reverse)	AGGTTGGTTAGGCAGGCACCTTT

© 2013 by the authors; licensee MDPI, Basel, Switzerland. This article is an open access article distributed under the terms and conditions of the Creative Commons Attribution license (http://creativecommons.org/licenses/by/3.0/).
